# Association of lymphocyte subsets and cytokines with bone metabolism: a retrospective, cross-sectional study

**DOI:** 10.1186/s12891-023-07137-8

**Published:** 2024-01-09

**Authors:** Cong Peng, Qiao Yang, Xiangrui Kong, Zhengzhong Sun, Liang Wang, Li Xiao

**Affiliations:** 1 Institute of Respiratory and Critical Medicine/Beijing Key Laboratory of OTIR, the Eighth Medical Center of PLA General Hospital, 17# Heishanhu Road, Haidian District, Beijing, 100091 China; 2 Department of Geriatrics, the Eighth Medical Center of PLA General Hospital, 17# Heishanhu Road, Haidian District, Beijing, 100091 China; 3 Hebei North University, Zhangjiakou, 075000 Hebei China; 4 Department of General Medicine and Geriatrics, Chongqing Emergency Medical Center, Chongqing University Central Hospital, Chongqing, 400010 China

**Keywords:** Bone metabolism, Immune system, Lymphocyte subset, Cytokine, Bone turnover marker

## Abstract

**Background:**

Previous research has shown that lymphocytes and cytokines can mediate bone metabolism. This study explored the clinical association and predictive ability of lymphocytes and cytokines levels for bone metabolism.

**Methods:**

A total of 162 patients were enrolled in this study. The levels of N-terminal propeptide of type I procollagen (P1NP), β-collagen degradation product (β-CTX), total T lymphocytes, immature T lymphocytes, suppressor/cytotoxic T lymphocytes, helper/inducer T lymphocytes, B lymphocytes, natural killer (NK) cells, Interferon-gamma (IFN-γ), tumour necrosis factor-alpha (TNF-α), IFN-α, interleukin-1 beta (IL-1β), IL-2, IL-4, IL-5, IL-6, IL-8, IL-10, and IL12p70 were evaluated. The relationship between these lymphocyte subsets and cytokines with bone metabolic status was examined and their predictive ability for bone metabolic status was assessed.

**Results:**

The principal component analysis (PCA) and correlation analysis results varied on differences in lymphocyte subsets and cytokines in various bone metabolism states. Differential analysis revealed significant differences in the absolute counts of B lymphocytes (*P* < 0.05), level of IL-12p70 (*P* < 0.05), and IL-8 (*P* < 0.001) at different P1NP levels. Significant differences were observed in the absolute counts of total T lymphocytes (*P* < 0.05), B lymphocytes (*P* < 0.05), the level of IL-6 (*P* < 0.05), the percentage of B lymphocytes (*P* < 0.01), and NK cells (*P* < 0.05) at different β-CTX levels. Furthermore, the receiver operating characteristic (ROC) curve showed that the absolute count of B lymphocytes and levels of IL-12p70 and IL-8 could be used to evaluate bone formation states, while the absolute counts of T and B lymphocytes, level of IL-6, and percentages of NK cells and B lymphocytes could be used to evaluate bone resorption states.

**Conclusion:**

The bone metabolism status changed based on the lymphocyte subsets and cytokine levels. Differentially expressed lymphocytes and cytokines could be used to distinguish bone metabolism status.

**Supplementary Information:**

The online version contains supplementary material available at 10.1186/s12891-023-07137-8.

## Background

Bone metabolic diseases are caused by a disruption in the metabolic balance between osteoblasts and osteoclasts [1]. The diagnosis and prognosis of bone metabolism disorders primarily rely on the assessment and analysis of bone mineral density using dual-energy X-ray absorptiometry (DEXA) [2, 3]. However, These tools present hysteresis in their evaluation of bone metabolism. The current evaluation methods for bone metabolism and its prognosis fall significantly short of meeting practical needs in terms of their evaluation and predictive capabilities. Indeed, these tools underestimate the risk of bone metabolic disorders and associated fractures and account for only a fraction of the total fracture risk [4–6]. Therefore, observing and predicting bone metabolism more accurately is necessary to manage fractures and other diseases associated with bone metabolic disorders [7].

Since bone metabolic disorders are strongly associated with the balance between osteoblasts and osteoclasts, biomarkers reflecting the activity of these cells can potentially reflect the current level of bone metabolism [8]. For example, the N-terminal propeptide of type I procollagen (P1NP) detects bone formation states [9, 10], while the β-collagen degradation product (β-CTX) reflect bone resorption states [11, 12]. These bone turnover markers have therefore been utilized for the diagnosis and management of various diseases, such as osteoporosis, Paget's disease, fibrous dysplasia, hypophosphatemia, primary hyperparathyroidism, and chronic kidney disease-mineral bone disease [13], as well as in assessing and predicting treatment response, bone mass, and fracture risk alongside other indicators of bone metabolism [8, 14]. However, differences in biological variability of individuals affect the accuracy of bone turnover markers [15, 16], and, in disease state, such as dermatological disorders and hepatic fibrosisbone, turnover markers result in diminished assessment efficiency [17, 18]. This ultimately affects the diagnostic and predictive ability of these bone turnover markers.

Therefore, it is imperative to explore new indicators capable of comprehensively reflecting bone metabolism, such as within the realm of osteoimmunology, to enhance the prediction and evaluation of bone metabolic disorders, as well as the effective management of adverse outcomes in patients with such conditions. Osteoimmunology focuses on the interaction between the immune system and bones [19] and explores bone metabolism from the intersection of these two fields.

Studies have found that the ratio of lymphocytes to neutrophils, monocytes, and platelets can indicate osteoporosis [20–22]. Circulating T lymphocyte subsets can predict bone morphology [23]. We have previously demonstrated a significant correlation between total T lymphocytes, CD8^+^ T lymphocytes, and bone mineral density (BMD) [24]. The findings suggest that lymphocytes and their secreted cytokines may serve as potential biomarkers for predicting and evaluating bone metabolism and its prognosis. Therefore, in this study, we analysed lymphocyte subsets, cytokines, and bone metabolism indicators (P1NP and β-CTX) in inpatients at our medical centre, and determined the correlations among these indicators to identify new potential indicators for the evaluation and prediction of bone metabolism.

## Methods

### Study design and ethics statement

This retrospective cross-sectional study was conducted at the Department of Geriatrics and Institute of Respiratory and Critical Medicine, Eighth Medical Center, PLA General Hospital following the ethical standards of the Declaration of Helsinki. The Ethics Committee of the Eighth Medical Center of PLA General Hospital approved this study (approval number: 309202109091005). All participants were aware of the study objectives and content and provided signed informed consent.

### Study participants and their basic characteristics

This study included 162 patients hospitalized in the Department of Geriatrics between December 23, 2021, and May 18, 2022. The inclusion criteria were as follows: patients over the age of 40 years with disorders of bone metabolism and immune system, most of which occurred above the age of 40. The exclusion criteria were as follows: (1) primary immune dysfunctional disease, severe infection, acquired immune deficiency, or allogeneic blood transfusion within 3 months; (2) use of immunomodulatory agents and hormone treatment within 1 year; (3) malignant tumours, major organ failure, or organ transplantation; (4) history of orthopaedic diseases characterized by bone damage; (5) other diseases that cause abnormal bone metabolism and immune regulation. General information about each participant, including the age, sex, height, weight, and body mass index (BMI), was collected. The medical history of each participant was recorded. Informed consent was obtained from the participants for the collection of these data.

### Detection of lymphocyte subsets

At admission, a 2-mL blood sample was taken from all participants in a fasting state. The samples were analysed using gating lymphocytes by CD45 staining versus side scatter (SSC), including total T lymphocytes (CD3^+^ CD45^+^), immature T lymphocytes (CD4^+^CD8^+^), suppressor/cytotoxic T lymphocytes (CD3^+^CD8^+^), helper/inducer T lymphocytes (CD3^+^CD4^+^), B lymphocytes (CD3^−^CD19^+^), and natural killer (NK) cells (CD3^−^CD16^+^CD56^+^) [25, 26].

First, 8 μL mixed antibodies (BD Biosciences, San Jose, CA, USA, No. 66299) were added to the bottom of absolute counter tubes (BD Multitest 6-Color TBNK Reagent, No. 662967), including CD45 (APC-Cy5.5), CD3 (FITC-A), CD4 (PE-Cy7-A), CD8 (APC-Cy7-A), CD19 (APC-A), and CD16CD56 (PE-A). Next, 50 μL of the blood sample was added to the bottom of the absolute counter tubes using a reverse pipette before vortex mixing. The mixture was incubated at room temperature (25 °C ± 1 °C) in the dark for 15 min. Subsequently, 450 μL of haemolysin (BD Biosciences, No. 349202) (FACS Lysing solution:distilled water = 1:10) was added to each tube, mixed well, and incubated at room temperature in the dark for 15 min. Finally, the absolute counts of the lymphocyte subsets were analysed and obtained using the FACSCanto Plus flow cytometer (BD Biosciences), and the ratio of each cell was calculated.

### Detection of cytokines

The blood samples were centrifuged (1,000 × *g* for 10 min) to collect plasma within 4 h after collection and stored at –20 °C until use. The levels of interferon-gamma (IFN-γ), tumour necrosis factor-alpha (TNF-α), IFN-α, interleukin-1 beta (IL-1β), IL-2, IL-4, IL-5, IL-6, IL-8, IL-10, IL-12p70, and IL-17 were determined using the 12 cytokines detection kit (multiple microglobulin immunofluorescence luminescence, China Qingdao Riskell Biotechnology Co., LTD.).

First, 25 μL of plasma, experimental buffer, capture microsphere antibody (RAISECARE, Qingdao, China, No. R701002), and detection antibody (RAISECARE, No. R701002) were sequentially added to the sample tube and incubated at room temperature while shaking for 2 h (400–500 rpm) in the dark. Subsequently, 25 μL of Streptavidin Phycoerythrin (SA-PE) (RAISECARE, No. R701002) was added to the tube and incubated at room temperature while shaking for 30 min (400–500 rpm) in the dark. Then, 1 mL washing buffer was added to the tube and vortexed at 1,500 r for 5 min. Next, the supernatant liquid was slowly poured out, and 70 μL washing buffer (RAISECARE, No. R701002) was added. Finally, the levels of cytokines were obtained using the FACSCanto Plus flow cytometer (BD Biosciences) and the test results were acquired using LEGENDplex Data Analysis software (v8.0).

### Detection of bone turnover markers

The plasma samples for bone turnover marker assays were prepared as for cytokine assays except that they were stored at –80 °C until use. The P1NP level was detected using a P1NP detection kit (Roche Diagnostics, Switzerland, No. 03141071–190), while the β-CTX content was detected using a serum-CTX detection kit (Roche Diagnostics, No. 11972308–122). Both assays are chemiluminescence immunoassays. According to the manufacturer's instructions, the reaction solution and plasma samples were aspirated into the measurement cell of a fully automatic electrochemiluminescence analyser (Cobas e601, Roche Diagnostics), and the detection results of P1NP and β-CTX were automatically obtained. In addition, the assay performance was verified using the manufacturer's control samples before the assay, according to the manufacturer's instructions.

As the study population comprised inpatients in a single centre in China, the reference range of bone metabolism indicators was implemented following the "Expert Consensus on the Clinical Application of Biochemical Indicators of Bone Metabolism (2019)" promulgated in China [27]. The PINP reference range was 31.7–70.7 ng/mL for females, while the average was 21–78 ng/mL. The bone formation states were altered when P1NP exceeded the reference range. The reference range of β-CTX detection was as follows: (1) the mean value for premenopausal females was 0.299 ng/mL; (2) mean value for postmenopausal females was 0.556 ng/mL; (3) mean value in males aged 30–50 years was 0.3 ng/mL; (4) mean value for males aged 50–70 years was 0.304 ng/mL; (5) mean value for males aged > 70 years was 0.394 ng/mL. The bone resorption states were altered when the β-CTX level exceeded the 95% confidence interval.

### Statistical analysis

GraphPad Prism 9 software (San Diego, CA, USA) was used to process, analyse, and visualize the data, and *P* < 0.05 indicated a statistical difference. The numbers represent enumeration data; continuous variables not normally distributed are represented as quartiles [50% (25–75%)], whereas those normally distributed as mean ± standard deviation. Furthermore, normally distributed continuous variables were analysed using the analysis of variance, whereas abnormally distributed data were analysed using Kruskal–Wallis test.

The effects of lymphocyte subsets and cytokines on bone metabolism in participants with different bone metabolism profiles were analysed using the principal component analysis (PCA). The principal components with eigenvalues ​​ > 1 were selected. Subsequently, the eigenvalues ​​were compared with the mean of the corresponding principal components. Moreover, the correlation between lymphocyte subsets and cytokines in patients with bone metabolic disorders was analysed, and the differences in different bone metabolism conditions were explored.

Additionally, the partial receiver operating characteristic (ROC) package in Rstudio3.6.4 was used to evaluate the overall efficacy of differentially expressed lymphocytes and cytokines in distinguishing bone metabolic status. The ROC curve reflected the diagnostic ability of tested cells and factors. The area under the curve (AUC) was > 0.5, indicating that the test item had diagnostic ability. Statistical significance was set at *P* < 0.05. The ggplot2 package in Rstudio3.6.4 was used for visualization.

## Results

### Participant characteristics

In total, 162 participants over the age of 40 years (72.03 ± 14.48 years) were included in this study and categorized into Cohort 1 (grouped according to the P1NP level within the cohort) and Cohort 2 (grouped according to the β-CTX level within the cohort) based on the P1NP and β-CTX levels.

In Cohort 1, the participants were categorized into three groups according to their P1NP levels. The low bone formation states group (L-P1NP) included 48 individuals (71.15 ± 15.53 years), 22 males and 26 females; the normal bone formation states group (N-P1NP) included 100 individuals (72.54 ± 13.87 years), 49 males and 51 females; the high bone formation states group (H-P1NP) included 14 individuals (71.43 ± 15.92 years), 4 males and 10 females. Significant differences were observed in the P1NP (*P* < 0.001) and β-CTX (*P* < 0.001) levels among the three groups (Table 1), as well as in weight (*P* = 0.02) and BMI (*P* = 0.009) (Table 1). No significant differences were found in age, sex, or height among the three groups (Table 1).

**Table 1:** Clinical characteristics of the study participants (*n* = 162)^c^

**Characteristic**	**Total (*n* = 162)**	**Cohort 1**^**a**^				**Cohort 2**^**b**^			
		**L-P1NP (*n* = 48)**	**N-P1NP (*n* = 100)**	**H-P1NP (*n* = 14)**	***P***	**L-β-CTX (*n* = 37)**	**N-β-CTX (*n* = 89)**	**H-β-CTX (*n* = 36)**	***P***
**Basic characteristic**									
Male/ Female ^d^	75/87	22/26	49/51	4/10	0.356	1/36	55/34	19/17	< 0.001
Age (years)^e^	72.03 ± 14.48	71.15 ± 15.53	72.54 ± 13.87	71.43 ± 15.92	0.947	67.49 ± 13.46	75.47 ± 13.07	68.19 ± 16.77	0.010
Height (m^2^)^e^	1.65 ± 0.07	1.65 ± 0.08	1.65 ± 0.07	1.64 ± 0.06	0.458	1.61 ± 0.06	1.67 ± 0.07	1.67 ± 0.07	< 0.001
Weight (kg)^e^	62.67 ± 9.34	63.91 ± 9.12	62.7 ± 9	58.19 ± 11.73	0.020	59.23 ± 10.03	64.84 ± 10.03	60.83 ± 8.17	0.007
BMI^e^	22.82 ± 2.32	23.37 ± 2.14	22.75 ± 2.3	21.50 ± 2.67	0.009	22.91 ± 2.23	23.24 ± 2.44	21.7 ± 1.74	0.005
**Markers of bone turnover**									
P1NP^e^	48.00 ± 55.15	17.35 ± 2.54	47.33 ± 16.93	157.84 ± 137.83	< 0.001	25.62 ± 2.17	43.67 ± 23.79	81.68 ± 103.17	< 0.001
β-CTX ^e^	0.50 ± 0.53	0.30 ± 0.20	0.48 ± 0.24	1.34 ± 1.44	< 0.001	0.19 ± 0.08	0.43 ± 0.14	1.00 ± 0.94	< 0.001

^a^This cohort was divided into low (L-P1NP), normal (N-P1NP), and high (H-P1NP) activity groups according to the reference range of P1NP

^b^This cohort was divided into low (L-β-CXT), normal (N-β-CXT), and high (H-β-CXT) activity groups according to the reference range of β-CXT

^c^Data represent mean ± SD or number

^d^Differences were compared using the chi-square test

^e^Differences were compared using the Mann–Whitney *U* test

In Cohort 2, participants were categorized into three groups according to the β-CTX level. The low bone resorption states group (L-β-CTX) c included 37 individuals (67.49 ± 13.46 years), 1 males and 36 females; the normal bone resorption states group (N-β-CTX) included 89 individuals (75.47 ± 13.07 years), 55 males and 34 females; the high bone resorption states group (H-β-CTX) included 36 individuals (68.19 ± 16.77 years), 19 males and 17 females. The results demonstrated that age (*P* = 0.010), sex (*P* < 0.001), weight (*P* = 0.007), height (*P* < 0.001), BMI (*P* = 0.005), P1NP level (*P* < 0.001), and β-CTX level ​​(*P* < 0.001) significantly differed among the groups (Table 1).

### Effect of different measures on bone metabolism

**Fig. 1:**
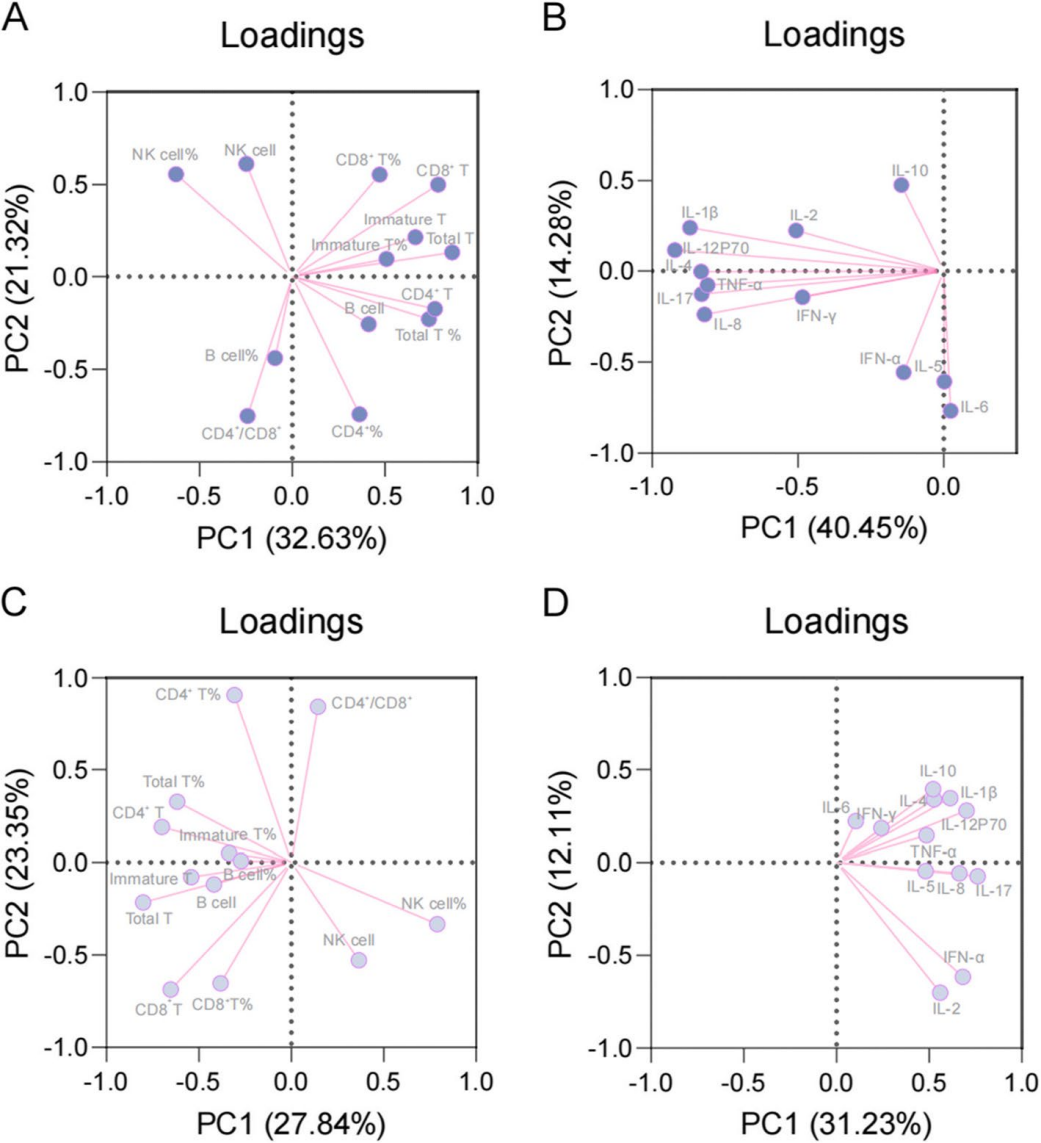
Principal component analysis of lymphocytes and cytokines in participants with different states of bone formation. The state of bone formation was categorized into disorder (A and B) and normal (C and D) groups according to the reference range of P1NP. The cumulative loading of PC1 and PC2 was 53.95% in the lymphocyte subsets of the disorder group **A** The cumulative loading of PC1 and PC2 was 55.73% in the cytokines of the disorder group **B** The cumulative loading of PC1 and PC2 was 51.39% in the lymphocyte subsets of the normal group **C** The cumulative loading of PC1 and PC2 was 43.33% in the cytokines of the normal group **D** P1NP, propeptide of type I procollagen

**Fig. 2:**
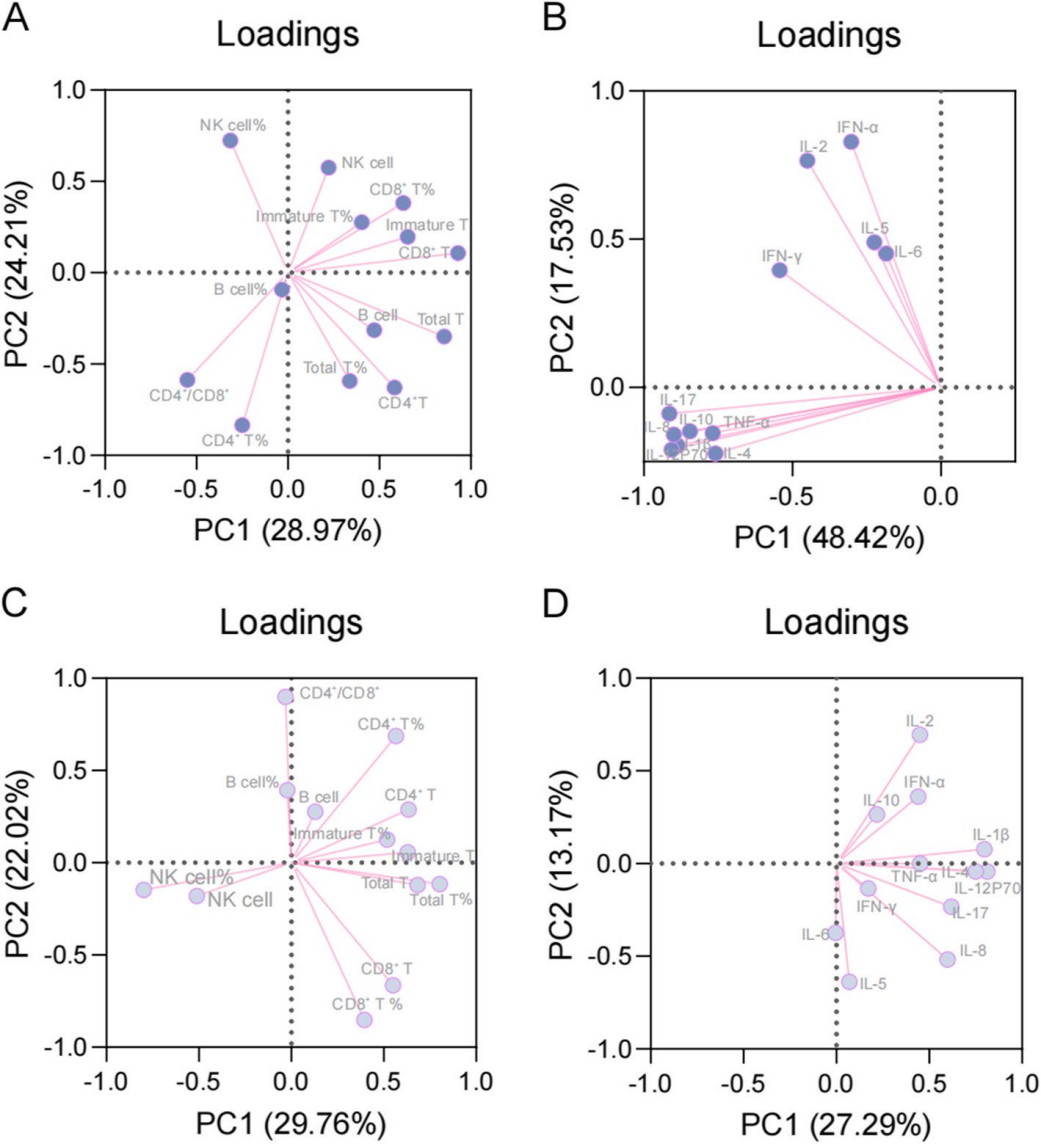
Principal component analysis of lymphocytes and cytokines in participants with different states of bone resorption. The state of bone resorption was categorized into disorder (A and B) and normal (C and D) groups according to the reference range of β-CXT. The cumulative loading of PC1 and PC2 was 53.18% in the lymphocyte subsets of the disorder group **A** The cumulative loading of PC1 and PC2 was 65.95% in the cytokines of the disorder l group **B** The cumulative loading of PC1 and PC2 was 51.78% in the lymphocyte subsets of the normal group **C** The cumulative loading of PC1 and PC2 was 40.46% in the cytokines of the normal group **D** β-CTX: beta-isomer of the C-terminal telopeptide of type I collagen

Effects of different measures on bone metabolic states, including absolute counts and percentage of lymphocyte subsets, were evaluated; all cytokines tested were included in the analysis. According to the reference ranges of P1NP and β-CXT, the bone metabolism status was divided into the bone formation disorder (*n* = 62) (Fig. 1A and B) and bone formation normal (*n* = 100) (Fig. 1C and D), as well as bone resorption disorder (*n* = 73) (Fig. 2A and B) and bone resorption normal (*n* = 89) (Fig. 2C and D).

The PCA showing the correlation between these measurements and the measurement with the highest eigenvalue for each principal component was generated. The absolute counts of total T lymphocytes and CD4^+^/CD8^+^ cells were associated with principal component 1 (PC1) (32.63%) and PC2 (21.32%), respectively (Fig. 1A). The levels of IL-12p70 and IL-6 correlated with PC1 (40.45%) and PC2 (14.28%), respectively (Fig. 1B). The absolute count of total T lymphocytes and percentage of CD4^+^ T lymphocytes correlated with PC1 (27.84%) and PC2 (23.35%), respectively (Fig. 1C). The levels of IL-17 and IL-2 correlated with PC1 (31.23%) and PC2 (12.11%), respectively (Fig. 1D). Furthermore, the absolute counts of CD8^+^ T lymphocytes and percentage of CD4^+^ T lymphocytes correlated with PC1 (29.87%) and PC2 (24.21%), respectively (Fig. 2A). The levels of IL-17 and IFN-α were associated with PC1 (48.42%) and PC2 (17.53%), respectively (Fig. 2B). The absolute counts of total T lymphocytes correlated with CD4^+^/CD8^+^ cells in PC1 (29.76%) and PC2 (22.02%) (Fig. 2C). The levels of IL-12p70 and IL-2 were associated with PC1 (27.29%) and PC2 (13.17%), respectively (Fig. 2D).

### Correlation analysis of lymphocyte subsets and cytokines in different bone metabolism status

**Fig. 3:**
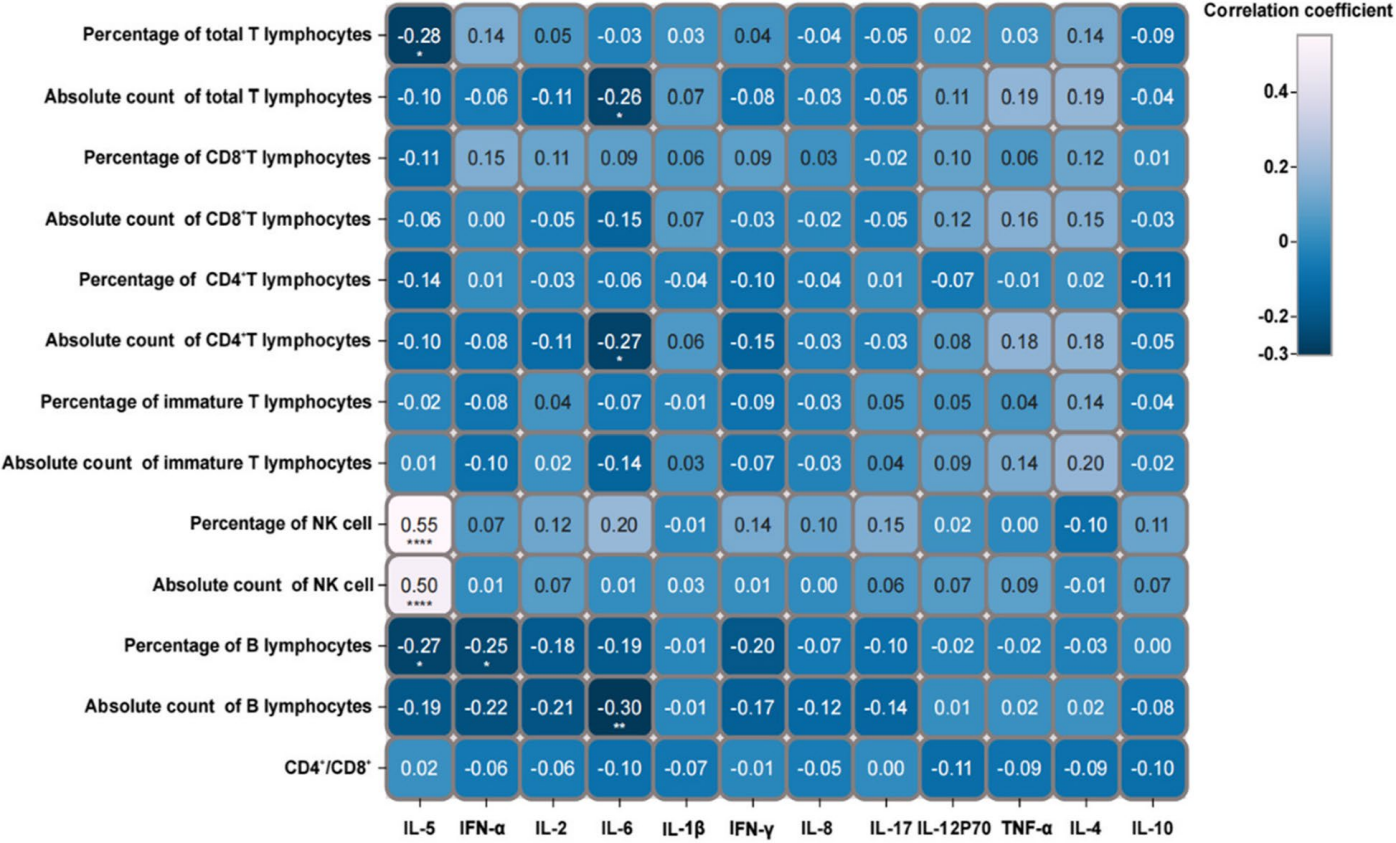
Correlation analysis of lymphocyte subsets and cytokines in participants with bone formation disorders. The results of the correlation analysis between lymphocyte subsets and cytokines in participants with bone formation disorders are displayed in a matrix diagram. The magnitude of the correlation coefficient is indicated in blue. The darker the colour, the higher the positive correlation, and the lighter the colour, the higher the negative correlation. Statistical significance is indicated as follows: ***P < 0.001;**P < 0.01; *P < 0.05

The correlation analysis between lymphocyte subsets and cytokines in bone formation disorder showed that the level of IL-5 correlated with the percentage of total T lymphocytes (R^2^ = -0.28, *P* < 0.05), percentage of NK cells (R^2^ = 0.55, *P* < 0.001), absolute count of NK cells (R^2^ = 0.50, *P* < 0.001), and percentage of B lymphocytes (R^2^ = -0.28, *P* < 0.05) (Fig. 3). The level of INF-α correlated with the percentage of B lymphocytes (R^2^ = -0.25, *P* < 0.05) (Fig. 3). The level of IL-6 correlated with the absolute count of total T lymphocytes (R^2^ = -0.26, *P* < 0.05), CD4^+^T lymphocytes (R^2^ = -0.27, *P* < 0.05), and B lymphocytes (R^2^ = -0.30, *P* < 0.01) (Fig. 3).

**Fig. 4:**
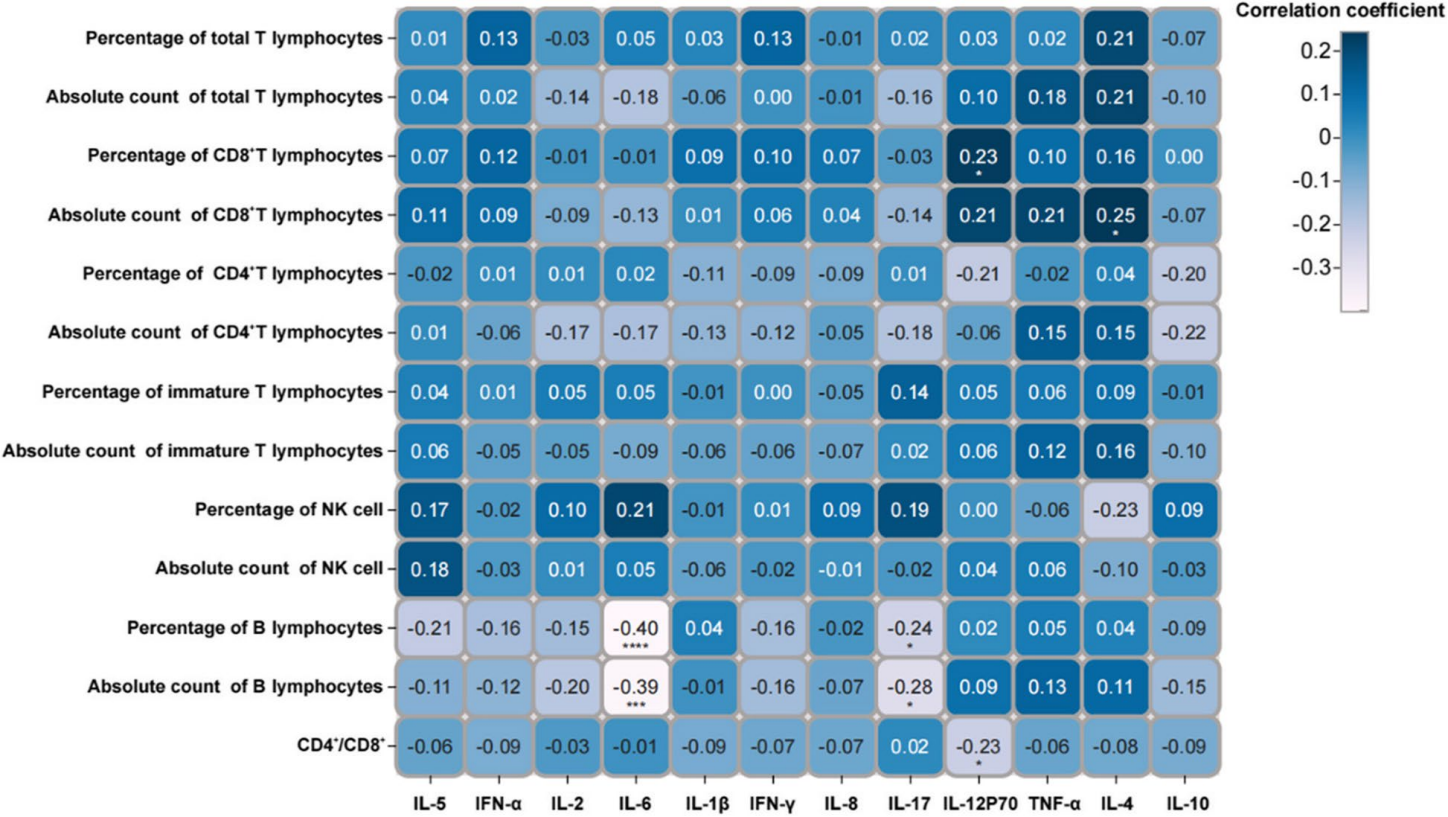
Correlation analysis of lymphocyte subsets and cytokines in participants with bone resorption disorders. The results of the correlation analysis between lymphocyte subsets and cytokines of participants with bone resorption disorders are displayed in a matrix diagram. The magnitude of the correlation coefficient is indicated in blue. The darker the colour, the higher the positive correlation, and the lighter the colour, the higher the negative correlation. Statistical significance is indicated as follows: ***P < 0.001;**P < 0.01; *P < 0.05

The correlation analysis between lymphocyte subsets and cytokines in bone resorption disorder showed that the level of IL-6 was significantly associated with the percentage of B lymphocytes (R^2^ = -0.40, *P* < 0.001) and absolute count of B lymphocytes (R^2^ = -0.39, *P* < 0.005) (Fig. 4). The level of IL-17 correlated with the percentage of B lymphocytes (R^2^ = -0.24, *P* < 0.05) and absolute count of B lymphocytes (R^2^ = -0.28, *P* < 0.05) (Fig. 4). The level of IL-12p70 correlated with the percentage of CD8^+^T lymphocytes (R^2^ = -0.40, *P* < 0.05) and CD4^+^/CD8^+^ (R^2^ = -0.39, *P* < 0.05) (Fig. 4). The level of IL-4 was associated with the absolute count of CD8^+^T lymphocytes (R^2^ = 0.25, *P* < 0.05) (Fig. 4).

### Effects of lymphocyte subsets and cytokines on bone metabolism

**Fig. 5:**
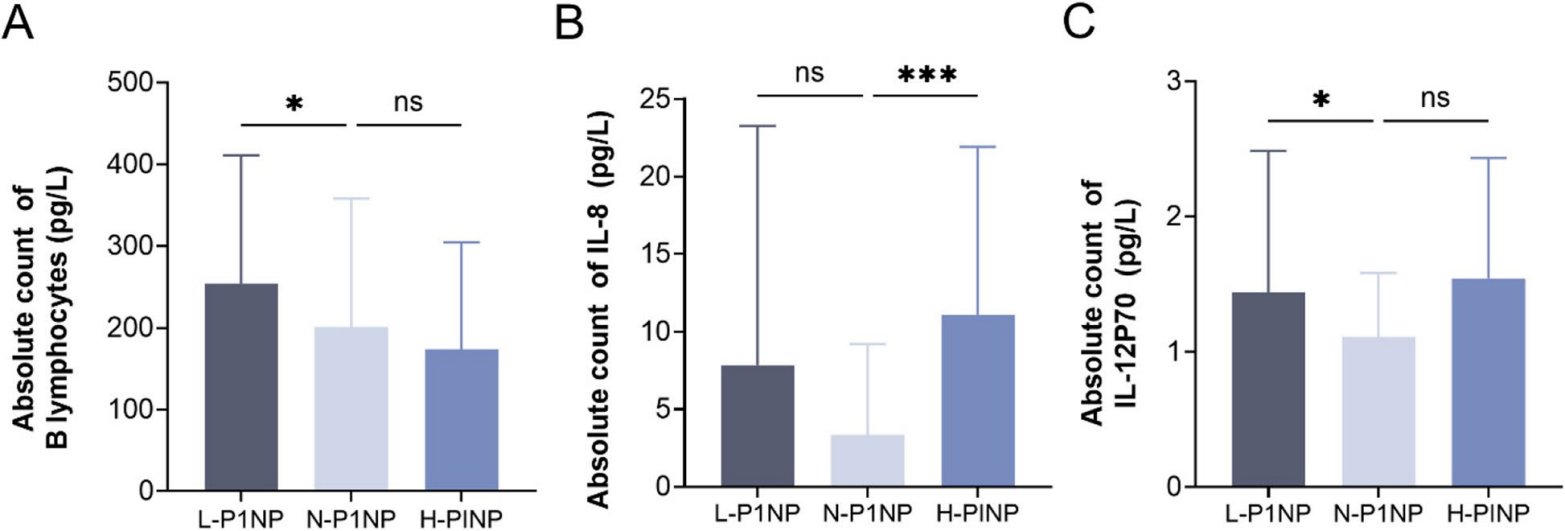
Comparison of the lymphocyte subsets and cytokines in cohorts with different bone formation states. The participants were categorized into low (L-P1NP), normal (N-P1NP), and high (H-P1NP) activity groups according to the reference range of P1NP. The absolute counts of B lymphocytes **A** and level of IL-12p70 **C** in the N-P1NP group were significantly lower than those in the L-P1NP group. The level of IL-8 **B** in the N-P1NP group was significantly lower than that in the H-P1NP group. All data were compared using Kruskal–Wallis tests and are shown as the mean ± SD. Statistical significance is indicated as follows: ***P < 0.001;**P < 0.01; *P < 0.05. P1NP, propeptide of type I procollagen

The Kruskal–Wallis test analysis showed that the absolute count of B lymphocytes (*P* < 0.05, Fig. 5A) and level of IL-12p70 (*P* < 0.05, Fig. 5C) in the L-P1NP group were significantly higher than those in the N-P1NP group. The level of IL-8 in the H-P1NP group (*P* < 0.001, Fig. 5B) was significantly higher than that in the N-P1NP group. However, no significant differences were observed in the levels of other lymphocytes or cytokines between the P1NP groups.

**Fig. 6:**
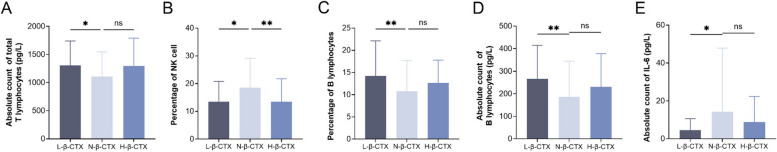
Comparison of the lymphocyte subsets and cytokines in cohorts with different bone resorption states. The participants were categorized into low (L-β-CXT), normal (N-β-CXT), and high (H-β-CXT) activity groups according to the reference range of β-CXT. The results demonstrated that the absolute count of total T lymphocytes **A** percentage of B lymphocytes **C** and absolute count of B lymphocytes **D** in the N-β-CXT group were significantly lower than those in the L-β-CXT group. The IL-6 **E** level in the L-β-CXT group was substantially lower than that in the N-β-CXT group. The percentage of NK cells **B** in the N-β-CXT group was significantly higher than that in the other two groups. All data were compared using Kruskal–Wallis tests and are shown as the mean ± SD. Statistical significance is indicated as follows: ***P < 0.001;**P < 0.01; *P < 0.05. β-CTX: beta-isomer of the C-terminal telopeptide of type I collagen

Additionally, the absolute count of total T lymphocytes (*P* < 0.05, Fig. 6A) and B lymphocytes (*P* < 0.01, Fig. 6D) and the percentage of B lymphocytes (*P* < 0.01, Fig. 6C) of the L-β-CTX group were significantly higher than those of the N-β -CTX group. The percentage of NK cells (*P* < 0.05, Fig. 6B) and level of IL-6 (*P* < 0.05, Fig. 6E) in the L-β-CTX group were significantly lower than those in the N-β-CTX group. The percentage of NK cells in the H-β-CTX group (*P* < 0.01, Fig. 6B) was significantly lower than that in the N-β-CTX group. However, no significant differences were observed in the other lymphocytes and cytokines among the β-CTX groups.

### Contribution of lymphocyte subsets and cytokines to the evaluation of bone metabolism

**Fig. 7:**
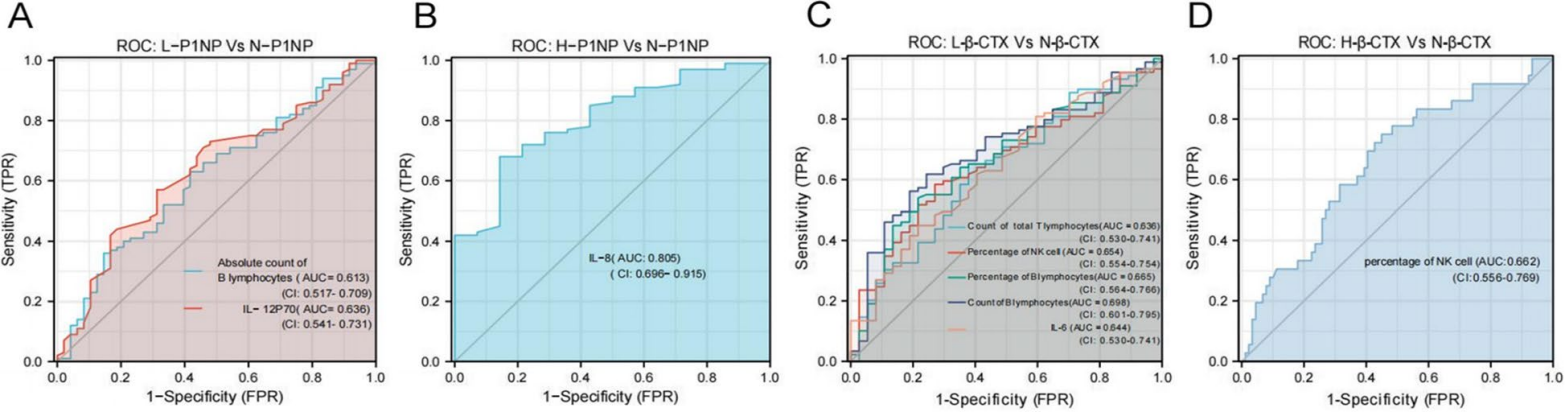
Analysis of the potential of lymphocyte subsets and cytokines to diagnose the bone metabolism condition. Receiver operating characteristic (ROC) curves were generated to determine whether lymphocyte subsets and cytokines can be used to diagnose the bone metabolism status. The results showed that the absolute count of B lymphocytes and level of IL-12p70 could distinguish between normal (N-P1NP) and low (L-P1NP) bone formation states **A** The level of IL-8 could differentiate between normal (N-P1NP) and high (H-P1NP) bone formation states **B** The absolute count of total T and B lymphocytes and percentage of B lymphocytes and NK cells could distinguish between normal (N-β-CXT) and high (L-β-CXT) bone resorption states **C** The percentage of NK cells could differentiate between normal (N-β-CXT) and low (H-β-CXT) bone resorption states **D** ROC curves were analysed to calculate the sensitivity and specificity of diagnostic accuracy

We determined whether lymphocyte subsets and cytokines could be used to evaluate the state of bone metabolism. The ability of differentially expressed lymphocytes and cytokines to predict the different levels of P1NP and β-CTX in each group was analysed using ROC curves. The results indicated that the absolute count of B lymphocytes (AUC = 0.613, *P* = 0.026) and level of IL-12p70 (AUC = 0.636, *P* = 0076) could be used to evaluate the reduction in the P1NP level. The level of IL-8 (AUC = 0.805, *P* = 0.0002) could be used to evaluate the r elevation in the P1NP level. Additionally, the absolute count of total T lymphocytes (AUC = 0.636, *P* = 0.0168) and B lymphocytes (AUC = 0.698, *P* = 0.0005), level of IL-6 (AUC = 0.644, *P* = 0.0112), and percentage of NK lymphocytes (AUC = 0.654, *P* = 0.0066) and B lymphocytes (AUC = 0.665, *P* = 0.0036) could be used to evaluate decrease in β-CTX level. The percentage of NK lymphocytes (AUC = 0.662, *P* = 0.0045) could be used to evaluate the elevation in the β-CTX level (Fig. 7, Table 2).

**Table 2:** Analysis of the diagnostic value of lymphocyte subsets and cytokines for abnormal bone metabolism by ROC curve^a^

Model	Cut-off value ^b^	Sensitivity	Specificity	positive predictive value	negative predictive value	Youden index ^c^	*P*
**Ability to differentiate L-P1NP from N-P1NP**							
Count of B lymphocytes	126.195	0.360	0.854	0.837	0.390	0.214	0.0260
IL-12p70	1.100	0.570	0.688	0.792	0.434	0.257	0.0076
**Ability to differentiate H-P1NP from N-P1NP**							
IL-8	2.520	0.680	0.857	0.971	0.273	0.537	0.0002
**Ability to differentiate L-β-CTX from N-β-CTX**							
Count of total T lymphocytes	1207.970	0.652	6.022	0.806	0.426	0.273	0.0168
Percentage of NK cells	15.030	0.584	0.730	0.839	0.422	0.341	0.0066
Percentage of B lymphocytes	10.085	0.539	0.784	0.857	0.414	0.323	0.0036
Count of B lymphocytes	183.185	0.618	0.757	0.859	0.425	0.375	0.0005
IL-6	4.140	0.449	0.784	0.833	0.372	0.233	0.0112
**Ability to differentiate H-β-CTX from N-β-CTX**							
Percentage of NK cells	16.190	0.750	0.551	0.403	0.845	0.301	0.0045

^a^Receiver operating characteristic

^b^Cut-off value for judging test positive and negative

^c^Methods for assessing the authenticity of screening tests. The larger the index, the better the effect of the screening experiment, and the greater the authenticity

## Discussion

This study analysed the relationship between bone metabolism and the immune system and showed that the absolute count of total T lymphocytes and percentage of CD4^+^ T lymphocytes and the levels of IL-17 and IL-2 are one of the principal components mediating bone formation disorders. This aligns with previous findings showing the regulatory role of T cells and CD4^+^ T lymphocytes in osteoblast activity through the secretion of various cytokines (including TNF-α and RANKL) [28, 29]. Similarly, IL-2 and IL-17 can also inhibit osteogenic activity by regulating RANKL or inducing inflammatory activity [30, 31]. These factors collectively mediate the activity of osteoblasts. Interestingly, IL-17 can also directly induce the formation of osteoclasts or indirectly promote osteoclast activity by enhancing the expression of pro-resorptive factors [32, 33]. This demonstrates the complexity and heterogeneity of the immune system's regulation of bone metabolism. Figures 3 and 4 demonstrate the heterogeneity in the correlation of immune cells with cytokines in bone formation and bone resorption disorder. Additionally, we observed that the total T lymphocytes, IL-12p70, and IL-2 were the primary components mediating bone resorption disorders, and have been reported to mediate bone loss or osteoclast activity [34–36]. In summary, the emergence of these complex regulatory networks provides new possibilities for exploring and regulating bone metabolism.

To identify potential biomarkers for assessing bone metabolism, we explored the differentially expressed lymphocytes and cytokines and showed an increase in B lymphocytes and IL-12p70 that led to a decrease in P1NP levels. This was consistent with the conclusion of previous studies that B lymphocytes inhibit osteogenic differentiation [37]. We also observed a synergistic increase in IL-8 and P1NP levels. It is possible that IL-8 activated T lymphocytes, which further led to bone loss and increased compensatory bone formation [38]. However, previous studies have only reported that IL-12p70 could stimulate Th1 and Th2 cells to inhibit the formation of osteoclasts by secreting the IFN-γ, without discussing its relationship with decreased bone formation [39]. In addition, we observed that the increase of T and B lymphocytes led to the reduction in the β-CTX levels. T and B lymphocytes are involved in the induction of osteoclastic activity in various inflammatory and immunological diseases [40–42]. Notably, in a relatively stable state, T lymphocytes inhibit osteoclast formation [41, 43, 44]; similarly, B lymphocytes can inhibit osteoclast formation by secreting TGF-β or limiting bone resorption under certain pathological conditions [45, 46]. In this study, we found that a decrease in the level of IL-6 led to the reduction of the β-CTX levels. Reduced IL-6 levels may reduce osteoclast formation and β-CTX levels. The positive correlation between IL-6 and osteoclast activity has been previously confirmed [47]. Surprisingly, the increase and decrease in the percentage of NK cells were accompanied by an increase in the β-CTX levels; however, previous studies only reported that NK cells negatively affect osteoclasts [48]. Therefore, we intend to further expand the sample size in future studies to clarify this unexpected finding.

Finally, we analysed the diagnostic and evaluation capabilities of these differentially expressed lymphocytes and cytokines using the ROC curve.

The ROC curve is used in bone metabolism research to determine the diagnostic ability of tested factors. For example, chitinase 3-like protein is used to determine the occurrence and development of osteoporosis [49]. The IL-6 is used to assess the risk of occurrence of fragility fractures [50]. In this study, the absolute count of B lymphocytes and level of IL-12p70 could distinguish a reduction in the states of bone resorption, while IL-8 levels could determine the hyper states of bone resorption. Additionally, the absolute count of total T lymphocytes and the absolute count and percentage of B lymphocytes can distinguish a reduction in the o states of bone formation. Simultaneously, the percentage of NK cells can predict both the hyperactivity of osteoclasts and a decrease in the states of bone formation. These lymphocytes and cytokines are potential bone metabolic biomarkers to assess the bone metabolic status of patients and their response to therapy. Overall, this study explored the relationship among bone metabolism, lymphocyte subsets, and cytokines at the clinical level, providing new potential perspectives and tools for understanding and evaluating bone metabolism.

This study has some limitations. First, this study was conducted in a single centre, which may affect the generalizability of the results. Therefore, we intend to include larger samples and conduct multicentre studies in future studies. Second, this was a retrospective analysis, and the outcome may be affected by non-observational factors; therefore, we intend to design prospective research based on the results of this study to assess the predictive and evaluation capabilities of the identified indicators. Third, the bone metabolism index is subject to several confounding factors, and it can only reflect bone metabolism rather than the bone metabolism outcome. Therefore, in subsequent studies, we intend to include multiple evaluation indicators of bone metabolism activity, such as BMD. Finally, this study only involved participants from northern China. Bone metabolism status differs among populations worldwide [51].

## Conclusions

This study revealed a link between bone metabolism and the immune system and showed that the decreased P1NP levels were associated with increased absolute count of B lymphocytes and IL-12p70 levels. The increased P1NP levels were associated with increased IL-8 levels. Similarly, the reduced β-CTX levels were associated with decreased IL-6 levels, increased absolute count of T and B lymphocytes, increased percentage of B lymphocytes, and reduced percentage of NK cells. In addition, the absolute count of T lymphocytes, level of IL-12p70, level of IL-8, absolute count and percentage of B lymphocytes, and percentage of NK cells may be used to evaluate different bone metabolic s tatus. These findings suggest potential biomarkers for bone metabolic disease prevention and diagnosis.

### Supplementary Information

**Additional file 1.**

## Data Availability

All data generated or analysed during this study are included in this published article.

## Abbreviations

BMD: Bone mineral density
BMI: Body mass index
P1NP: Procollagen type I N-terminal propeptide
β-CTX: β-collagen degradation product
ROC: Receiver operator characteristic curve
AUC: Area under the curve
PCA: Principal component analysis
PC: Principal component score
PC1: Principal component 1
PC2: Principal component 2
RANK: Receptor activator for nuclear factor-κβ
RANKL: Receptor activator for nuclear factor-κβ ligand
IL: Interleukin
INF: Interferon
TNF: Tumour necrosis factor
